# The monitoring of emergent zoonotic pathogens in wild and captive birds in Sarajevo Canton, Bosnia and Herzegovina

**DOI:** 10.3389/fvets.2025.1621094

**Published:** 2025-07-23

**Authors:** Adis Softić, Melisa Nicevic, Amira Koro-Spahic, Ilma Terzic, Sejla Goletic, Naida Kapo, Jasna Salkic, Jasmin Omeragic, Teufik Goletic

**Affiliations:** ^1^Veterinary Faculty, University of Sarajevo, Sarajevo, Bosnia and Herzegovina; ^2^Department of Pathology, University Clinical Center, Tuzla, Bosnia and Herzegovina; ^3^Faculty of Medicine, University of Tuzla, Tuzla, Bosnia and Herzegovina

**Keywords:** birds, emergent zoonotic diseases, public health, *C. psittaci*, WNV

## Abstract

**Introduction:**

With their remarkable flight capabilities, wild and captive birds play a pivotal role in the global dissemination of zoonotic pathogens including *Chlamydia psittaci*, Avian Influenza viruses (AIV), Chikungunya virus (CHIKV), Usutu virus (USUV), and West Nile virus (WNV). They function both as hosts and reservoirs responsible for transporting the mentioned infectious agents across vast geographic regions. Additionally, captive birds and birds inhabiting urban environments, particularly in tourist destinations, present significant public health concerns due to facilitated close interactions with humans.

**Methods:**

A total of 358 samples originating from fifteen bird species were collected across 21 locations in Sarajevo Canton, over three consecutive years (2022–2024). Upon collection, the samples were subjected to molecular analysis to detect the presence of zoonotic pathogens. For detection of *Chlamydia* spp., and *C. psittaci*, real-time PCRs (qPCR) were used following established protocols. Additionally, reverse transcriptase real-time PCR (RT-qPCR) were utilized for the detection of emergent viral pathogens including avian influenza viruses, Chikungunya, Usutu, and West Nile virus.

**Results:**

*Chlamydia* spp. was detected in 29.9% (95% CI: 25.2–34.9) of samples. Further, *C. psittaci* was identified in 10.3% (95% CI: 5.2–17.7) of positive samples originating from captive birds and birds inhabiting urban environments. One sample (0.3%) originating from a wild bird was positive to West Nile Virus. None of the samples tested positive for Avian Influenza viruses, Chikungunya and Usutu virus.

**Discussion:**

The identification of *C. psittaci* and West Nile virus highlights the increased likelihood of zoonotic transmission. This underscores the imperative for bolstered biosecurity measures and public health strategies aimed at mitigating the risk associated with both environmental exposure and direct contact, especially in areas characterized by substantial tourist activity.

## 1 Introduction

Birds—encompassing migratory, urban, and captive species—play a crucial role in the transmission dynamics of zoonotic pathogens (1, 2). Their significance to public health is underscored by their ability to serve as both hosts and vectors for a variety of infectious agents with zoonotic potential. The capacity of birds to harbor and disseminate pathogens enables transmission across diverse environments (3, 4). Urban-adapted birds, such as feral pigeons (*Columba livia domestica*), often function as reservoirs for *Chlamydia psittaci*—the agent of avian chlamydiosis (psittacosis) in humans (4–6). *C. psittaci* is an obligatory intracellular bacterium known to infect a wide range of avian hosts as well as some mammals (6, 7). Infected birds typically shed the bacterium in feces and in ocular or respiratory secretions; transmission to other animals and humans occurs mainly via inhalation of contaminated dust or aerosols from dried droppings, feathers, or secretions (8). Once shed material dries, *C. psittaci* can remain viable in the environment for months, posing challenges for outbreak control and emphasizing the need for robust surveillance (7, 9). Birds often shed the pathogen asymptomatically, complicating detection and increasing the risk of unrecognized human exposure (10). Psittacosis in humans can cause severe atypical pneumonia, and those in close contact with birds (e.g., pet owners, breeders, zookeepers) are at particularly elevated risk of infection (11). Moreover, birds residing in urban and tourist areas frequently interact with people, which elevates the chance of zoonotic transmission through direct contact or inhalation of contaminated dust (5). These factors make *C. psittaci* a notable public health concern in the human–bird interface.

In addition to *C. psittaci*, many bird species are carriers of emerging viral pathogens such as West Nile virus (WNV), Usutu virus (USUV), Chikungunya virus (CHIKV), and highly pathogenic avian influenza viruses (HPAIV). WNV and USUV are mosquito-borne flaviviruses (family Flaviviridae, genus Flavivirus) belonging to the Japanese encephalitis serocomplex (12). They are primarily transmitted among vertebrates via *Culex* mosquitoes, with *Culex pipiens* being a principal vector in Europe (2, 12, 13). WNV is a prominent emerging arbovirus that poses significant public health risks (14). Human WNV infection ranges from asymptomatic or mild febrile illness (West Nile fever) to severe neuroinvasive diseases such as meningitis and encephalitis (12, 15, 16). In Europe, WNV has caused seasonal outbreaks in several countries, and predicting the location and intensity of WNV activity is challenging due to the influence of climate on mosquito populations (13). Birds are the primary amplifying hosts for WNV, and migratory species can carry the virus over long distances, complicating efforts to anticipate outbreaks (12, 13). USUV, a related flavivirus, has emerged in Europe over the past two decades and is now endemic in several countries (12, 16). In humans, USUV infection is usually mild (often causing fever and rash) but can lead to neuroinvasive illness in immunocompromised individuals (15, 16). CHIKV, by contrast, is an alphavirus (family Togaviridae) primarily transmitted by *Aedes* mosquitoes (especially *Ae. aegypti* and *Ae. albopictus*) rather than by birds (17, 18). Birds are not known reservoirs for CHIKV, but this virus is noteworthy as an emerging arbovirus that has caused outbreaks in temperate regions via human–mosquito transmission cycles (18, 19). Human chikungunya infection typically presents with high fever, arthralgia, and skin rash (17, 20). The spread of CHIKV into new areas of Europe is facilitated by the expanding range of *Aedes* vectors and suitable climate conditions, highlighting an ongoing public health concern even in regions where birds are not part of its cycle (18). Environmental and ecological factors strongly influence the transmission cycles of WNV, USUV, and other vector-borne viruses. Because these viruses involve bird–mosquito transmission, factors such as mosquito abundance, avian host species composition and immunity, and climate conditions for mosquito breeding can modulate outbreak risk (21, 22). Migratory birds in particular play a crucial role in the long-distance spread of flaviviruses and other pathogens, creating new foci of infection that can affect both wildlife and humans (2–4). A well-documented example of interspecies pathogen transmission by migratory birds is the global dissemination of HPAIV H5N1. Wild waterfowl carrying H5N1 along migratory routes have been implicated in introducing this virus to new regions (1). HPAIV H5N1 causes severe disease with high mortality in domestic and wild birds and can infect humans with often fatal outcomes (2, 23–25). While influenza A viruses cause significant respiratory outbreaks in multiple species (including humans, pigs, and horses), wild and domestic birds are central to the epidemiology of these viruses (1, 23). Waterfowl are considered primary reservoirs for all influenza A strains that affect mammals, and they can shed these viruses via the fecal-oral route while remaining asymptomatic, thereby perpetuating viral circulation in the environment (1, 23, 26).

This body of evidence highlights the importance of comprehensive surveillance of avian populations to mitigate zoonotic disease risks. In particular, a One Health approach—integrating wildlife surveillance with human and environmental health measures—is increasingly recognized as essential for early detection and control of emerging zoonoses. In line with these principles, the present study surveyed wild and domestic birds in Sarajevo Canton (Bosnia and Herzegovina) for the presence of *C. psittaci*, *Chlamydia* spp., WNV, USUV, CHIKV, and AIV using RT-qPCR assays. The goal was to generate baseline data on the circulation of these emergent zoonotic pathogens in urban and suburban regions.

## 2 Materials and methods

### 2.1 Sample collection

**Figure 1:**
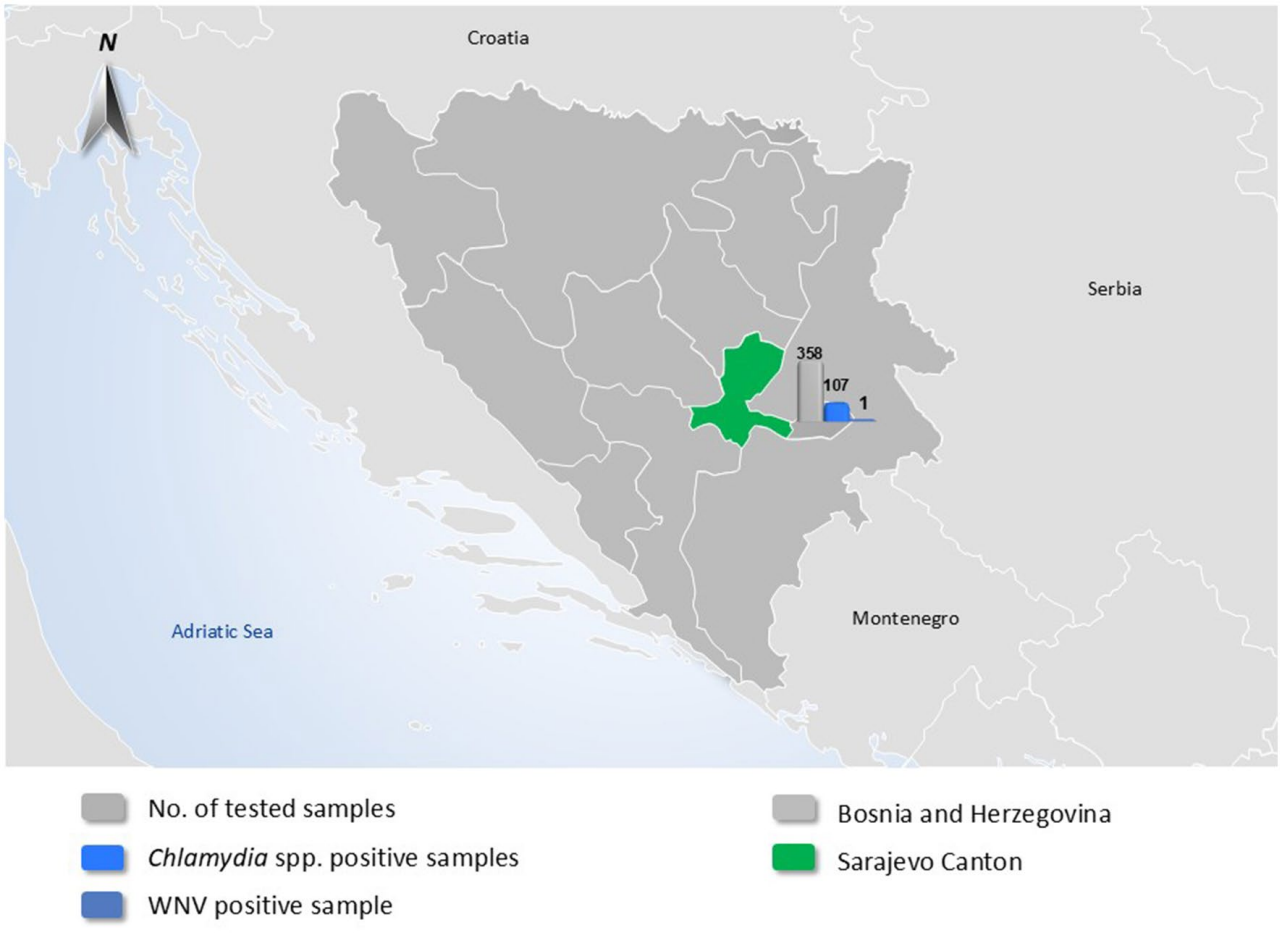
Geographic location of Sarajevo Canton and summary of pathogen detection in tested birds.

A total of 358 individual birds were collected from birds across twenty-one locations in Sarajevo Canton, Bosnia and Herzegovina, over a three-year period (2022–2024). The sampling included both domestic and wild birds. The sampling approach was non-random and opportunistic, primarily involving birds found dead, exhibiting signs of illness, or otherwise accessible through collaboration with local veterinary and environmental authorities. Sampling was conducted intermittently throughout the years 2022–2024, with most samples collected during spring, summer, and early autumn months when field activity and bird mortality were highest. To illustrate the geographic origin of the sampled birds and provide an overview of pathogen detection, a map of Bosnia and Herzegovina was generated. Sarajevo Canton, the sole sampling region, was highlighted in green. Overlaid bar charts summarize the total number of avian samples tested, as well as the number of samples positive for *Chlamydia* spp. and West Nile virus (WNV) (Figure 1). These visual elements were added to convey the spatial and quantitative distribution of the tested pathogens in a single visual format. Domestic birds sampled were chickens (*Gallus gallus domesticus*) and pigeons (*Columba livia domestica*), while wild bird species (13 species in total) were selected based on their known roles as hosts or amplifiers of zoonotic pathogens. The wild species sampled included Black-headed Gull (*Chroicocephalus ridibundus*), Common Gull (*Larus canus*), Common Blackbird (*Turdus merula*), Common Pheasant (*Phasianus colchicus*), Common Raven (*Corvus corax*), Hooded Crow (*Corvus cornix*), Western Jackdaw (*Corvus monedula*), Eurasian Magpie (*Pica pica*), Eurasian Chaffinch (*Fringilla coelebs*), House Sparrow (*Passer domesticus*), Rock Pigeon (*Columba livia* feral populations), Mute Swan (*Cygnus olor*), and Wild Duck (*Anas platyrhynchos*). Samples consisted of freshly deposited feces and, in some cases, tissues from bird carcasses found on the day of sampling. Fecal samples were collected using sterile swabs and placed in transport medium. Cloacal and fecal swabs were selected for avian influenza virus (AIV) detection, in line with the known fecal–oral transmission dynamics of influenza A viruses in birds. This specimen type is widely used in avian surveillance programs to detect viral shedding in asymptomatic carriers. Carcasses of wild birds were transported to the laboratory on the day of collection; during necropsy, liver tissue and intestinal contents were aseptically collected. Liver and intestinal samples were selected based on their diagnostic utility for detecting both systemic and enteric pathogens under field conditions. Other tissues (e.g., brain, lungs, spleen) were not routinely collected due to biosafety considerations and practical limitations in necropsy infrastructure. All samples were transported from the field in insulated coolers with ice packs and maintained at approximately +4°C. Upon arrival at the laboratory, samples were either processed the same day or stored at −80°C until further molecular analysis. Liver samples were chosen for virological testing (for WNV, USUV, CHIKV, and AIV), whereas intestinal contents and feces were used for *Chlamydia* spp. screening. All bird handling and sampling procedures followed ethical guidelines for wildlife research and animal welfare, with efforts made to minimize disturbance to birds and their habitats. Sampling sites included urban parks, farms, wetlands, and other areas frequented by the target species, many of which are also visited by local residents and tourists.

### 2.2 Molecular analysis

All molecular work was performed in a Biosafety Level 2 (BSL-2) laboratory in accordance with applicable biosafety guidelines for the handling of zoonotic pathogens. Nucleic acids were extracted using commercial kits appropriate for the sample type: the QIAamp Viral RNA Mini Kit (Qiagen, Hilden, Germany) for RNA viruses and the QIAamp DNA Mini Kit or DNeasy Blood & Tissue Kit (Qiagen, Hilden, Germany) for DNA pathogens, following the manufacturers’ protocols. Tissue samples were homogenized using a TissueLyser II (Qiagen, Hilden, Germany) prior to extraction to ensure efficient recovery of pathogens. For detection of bacteria from the genus *Chlamydia*, we performed a qPCR targeting a conserved region of the *Chlamydia* 23S rRNA gene (27). All samples positive for *Chlamydia* spp. were subsequently tested with a species-specific qPCR to confirm *C. psittaci*, targeting the ompA gene (28). Both assays were carried out in a Stratagene Mx3005P thermal cycler (Agilent Technologies, Santa Clara, CA, USA) with appropriate positive controls (*C. psittaci* DNA) and negative controls included in each run. In parallel, we screened for four emergent viruses (AIV, WNV, USUV, and CHIKV) using previously published RT-qPCR protocols. Avian influenza A virus (AIV) RNA was detected with the highly conserved matrix-gene real-time RT-qPCR described by Heine et al. (29). Detection of WNV RNA was performed with primers and probe targeting the WNV *NS2A* gene region as previously described (30). USUV detection employed the real-time RT-qPCR assay developed by Cavrini et al. (31), targeting the USUV *NS5* gene, while CHIKV RNA was detected using the protocol established by Rezza et al. (19), targeting the CHIKV *E1* gene region. All protocols incorporated validated primer-probe sets and thermal-cycling conditions optimized for specificity and sensitivity (Supplementary Table S1). All molecular analyses were conducted at the Laboratory for Molecular-Genetic and Forensic Investigations, Veterinary Institute, University of Sarajevo - Veterinary Faculty.

## 3 Results

Over the three-year period, 358 avian samples (274 fecal swabs and eighty-four organ samples) were collected and analysed. The fecal samples were obtained from domestic chickens (35 samples), domestic pigeons (28), common gulls (11), common pheasants (11), common ravens (16), feral rock pigeons (69 samples), hooded crows (53), wild ducks (88), mute swans (12), peafowl (6), house sparrows (7), common blackbirds (5), and Eurasian magpies (10). Liver and intestinal samples from each carcass were processed and tested as described above.

Out of 358 total samples, *Chlamydia*-genus DNA was detected in 107 samples, yielding an overall *Chlamydia* spp. prevalence of 30.2% among the tested birds. All *Chlamydia*-positive results originated from fecal or intestinal samples, whereas none of the liver samples showed presence of *Chlamydia* DNA. The 107 *Chlamydia*-positive samples were distributed across nine of the sixteen bird species evaluated (56.3%). Notably, *Chlamydia* spp. DNA was detected in specimens from rock pigeons, feral domestic pigeons, hooded crows, common ravens, western jackdaws, Eurasian magpies, house sparrows, mute swans, and wild ducks. The locations with positive detections encompassed eight distinct public sites within Sarajevo Canton, commonly frequently visited by both residents and tourists. Rock pigeons had the highest prevalence: 52.2% (95% CI: 39.8–64.4) of sampled feral rock pigeons were positive for *Chlamydia* spp. Among the 107 *Chlamydia*-positive samples, 11 (10.3, 95% CI: 5.2–17.7) were further confirmed as *Chlamydia psittaci*. These *C. psittaci*–positive samples came from two categories of birds: urban feral rock pigeons and captive, domestically kept pigeons. In rock pigeons, the prevalence of *C. psittaci* was 27.8% (14.2–45.2) (combining fecal and intestinal samples) and in domestic captive pigeons it was 10% (2.5–44.5). No other bird species in our sample set tested positive for *C. psittaci*.

Regarding viral pathogens, only a single sample tested positive out of all 358 examined (0.3, 95%CI: 0.01–1.6). This was a liver sample from a wild hooded crow (*Corvus cornix*), which showed borderline positivity for WNV RNA by RT-qPCR (Cycle threshold value – 32.9). This positive crow was collected in late summer 2023. All other samples (including all tested chickens, waterfowl, and other species) were negative for WNV.

None of the samples showed detectable RNA of USUV, CHIKV, or AIV. Thus, the prevalence of USUV, CHIKV, and AIV was 0% in our sample set. Table 1 provides a consolidated overview of pathogen detection results by bird species, including the number of tested individuals, number and percentage of positives for *Chlamydia* spp., *C. psittaci*, and WNV. Results are grouped according to bird status (domestic, peridomestic/feral, wild). Only a single WNV-positive bird—a hooded crow—was detected, while *Chlamydia* spp. and *C. psittaci* were identified in several species, particularly among pigeons and wild ducks.

**Table 1:** Detection of *Chlamydia* spp., *Chlamydia psittaci*, and West Nile virus (WNV) in domestic and wild bird species sampled in Sarajevo Canton (2022–2024).

Species	*Chlamydia* spp.				*C. psittaci*				WNV			
	Test	Pos	% (CI)	Ct	Test	Pos	% (CI)	Ct	Test	Pos	% (CI)	Ct
Domestic birds												
Chicken	35	7	20 (8.4–36.9)	26.7–34.1	7	ND	–	–	35	ND	–	–
Pigeon	28	10	35.7 (18.6–55.9)	25.8–34.6	10	1	10 (2.5–44.5)	29.9	28	ND	–	–
Wild birds												
Common gull	11	1	9.1 (0.2–41.3)	29.1–33.2	1	ND	–	–	7	ND	–	–
Common pheasant	11	ND	–	–	ND	–	–	–	11	ND	–	–
Common raven	16	4	25 (7.3–52.4)	27.1–30.7	4	ND	–	–	16	ND	–	–
Rock pigeon	69	36	52.2 (39.8–64.4)	24.3–33.8	36	10	27.8 (14.2–45.2)	27.1–31.3	69	ND	–	–
Hooded crow	53	18	34 (21.5–48.3)	23.9–32.2	18	ND	–	–	53	1	1.8 (0.5–10.1)	32.9
Wild duck	88	26	29.6 (20.3–40.2)	24.0–33.3	26	ND	–	–	88	ND	–	–
Mute swan	12	4	33.3 (9.9–65.1)	25.4–28.9	4	ND	–	–	12	ND	–	–
Peafowl	6	ND	–	–	–	–	–	–	6	ND	–	–
House sparrow	7	ND	–	–	–	–	–	–	7	ND	–	–
Common blackbird	5	–	–	–	–	–	–	–	5	ND	–	–
Eurasian magpie	10	1	10 (0.3–44.5)	27.0	ND	–	–	–	10	ND	–	–
Western jackdaw	6	ND	–	–	–	–	–	–	6	ND	–	–
Black-headed gull	1	ND	–	–	–	–	–	–	1	ND	–	–
Total	358	107	29.9 (25.2–34.9)	–	107	11	10.3 (5.2–17.7)	–	358	1	0.3 (0.1–1.6)	–

No samples were positive to AI, USUT and CHIKV. Test, number of tested birds; Pos, number of positive birds; %(CI), prevalence and 95% confidence interval; Ct, cycle threshold value (min to max); ND, not detected.

## 4 Discussion

Our findings demonstrate that *Chlamydia psittaci* is circulating in the avian population of Sarajevo Canton, particularly among pigeons, and that WNV is present at least sporadically in local wild birds. These results have significant public health implications. Pigeons, being inherently social and often commensal with humans in urban settings, are well-recognized as reservoirs of *C. psittaci* and other pathogens (32). In our study, 27.8% of tested feral (wild) pigeons were positive for *C. psittaci*. This prevalence aligns closely with a prior study in neighboring Croatia, which reported *C. psittaci* in 15.8% of tested pigeons (33). It is also consistent with the global prevalence estimate of about 17% for *C. psittaci* in pigeons reported in a recent meta-analysis (32). The detection of *C. psittaci* in Sarajevo’s feral pigeons is particularly concerning given that many samples originated from heavily frequented urban locations. For example, Baščaršija (the old town tourist centre of Sarajevo) is known for its dense pigeon population, actively fed by residents and tourists as part of a local tradition. Such close contact between humans and large aggregations of pigeons creates an environment ripe for zoonotic transmission of *C. psittaci* through inhalation of contaminated dust or direct contact. In addition to wild pigeons, we found *C. psittaci* in 10% of captive (domestic) pigeons, specifically racing or homing pigeons kept by local breeders. Although the prevalence was lower than in feral birds, it still indicates that captive pigeons can be carriers of this zoonotic agent. Racing pigeons often have opportunities for contact with wild birds during training flights or competitions, which can lead to infection with *C. psittaci* and other pathogens. Infected pigeons, even if asymptomatic, pose a risk of transmitting psittacosis to their owners and to others who manage them (11). This underscores the need for pigeon fanciers to be aware of psittacosis and to practice good husbandry and hygiene (such as regular health checks for their birds and use of personal protective equipment when cleaning lofts) to reduce the risk of infection. Our data reinforce the notion that pigeons (both wild and domesticated) are important reservoirs for *C. psittaci* in urban environments and that they can serve as a bridge for disease transmission to humans, exemplifying a classic One Health scenario where animal health directly impacts human health. In addition to pigeons, *Chlamydia* genus DNA was also detected in 20.0% (7/35) of tested domestic chickens. Although these samples were not confirmed to harbor *C. psittaci*, the detection of *Chlamydia* spp. in backyard poultry raises potential concerns. Backyard or small-scale poultry flocks are increasingly common in peri-urban settings and may have limited veterinary oversight. While *C. psittaci* is the most recognized zoonotic species, other *Chlamydia* species (e.g., *C. gallinacea*) have been identified in chickens and may have zoonotic potential, though their pathogenicity remains under investigation (34). These findings suggest that chickens, like pigeons, could function as reservoirs or incidental hosts, underscoring the need for further species-level characterization and biosecurity awareness in smallholder poultry systems. According to the Communicable Disease Bulletins issued by the Institute of Public Health of Sarajevo Canton, a total of 18 cases of chlamydial infections were reported between 2022 and 2024 (35). However, these reports do not distinguish between urogenital infections caused by *Chlamydia trachomatis* and respiratory infections such as psittacosis caused by *C. psittaci*. This lack of specificity, combined with limited clinical awareness and diagnostic capacity for *C. psittaci*, suggests that psittacosis in humans may be underdiagnosed or misclassified. Given the proximity and frequent contact between humans and urban bird populations, especially pigeons, this underestimation represents a potential blind spot in current public health surveillance and highlights the need for enhanced clinical recognition and laboratory support.

In contrast to the high detection rate of *Chlamydia* spp. in birds, our screening for emergent viruses yielded mostly negative results, which is somewhat reassuring. One notable viral result from our study was the detection of WNV in a hooded crow sample from 2023. Although not the first confirmed case—WNV was previously detected in a wild crow in Bosnia and Herzegovina in 2013 (data not published)—our finding represents the first published evidence of WNV circulation in avian host in this region. This finding reinforces ongoing virus circulation and has important implications. Our finding is consistent with broader trends: widespread WNV activity in Europe in 2023. According to the European Centre for Disease Prevention and Control (ECDC) reports, multiple European countries (including Italy, Germany, Spain, Bulgaria, Hungary, Austria, Greece, and Croatia) experienced WNV outbreaks in the 2023 transmission season, with numerous human cases reported (36). Our detection of WNV in a local corvid corroborates the presence of the virus in this region during that period. Corvid birds are well-established sentinels and amplifiers for WNV. Species like crows and jays often develop elevated levels of viremia and suffer mortality from WNV, which is why their infection can signal virus activity in an area (22, 37, 38). The infected hooded crow in our study may have acquired WNV through mosquito bites, which are the primary transmission route for this virus in birds. However, other transmission pathways—including oral ingestion of infected prey, direct contact, or environmental exposure—have also been reported (39) and cannot be excluded in this case. While birds are the amplifying hosts that infect mosquitoes, most other vertebrates (including humans and horses) are dead-end hosts that do not contribute significantly to further transmission due to low viremia (13, 14). Nevertheless, the spillover of WNV from birds to humans is a critical concern. Bosnia and Herzegovina has reported human WNV infections in past years, predominately neuroinvasive cases during the 2013 outbreak (40), although surveillance in humans is sparse. Our bird surveillance adds evidence that the pathogen is present in the local ecosystem. Public health authorities should therefore be prepared for the possibility of human WNV cases and consider mosquito control and public education on reducing mosquito bites during the summer months. We did not detect any avian influenza virus, CHIKV, or USUV in the samples. The absence of CHIKV and USUV in these birds is not surprising: CHIKV is not typically associated with birds (its primary cycle is human–mosquito), and USUV, while bird-associated, tends to be focal in its distribution and might not have been present in our specific sample set or region during the study period. Although birds are not known to serve as amplifying hosts for CHIKV, the virus was included in the panel as part of an integrated arboviral surveillance approach, in line with One Health principles. However, the negative finding for USUV should be interpreted with caution. USUV has been confirmed to be circulating in several European countries, including our close neighbors Croatia and Serbia, in recent years (16). It is possible that USUV could emerge or be present at low levels in Bosnia and Herzegovina as well. While the presence of *C. psittaci* and WNV in avian populations indicates a potential zoonotic risk, it does not, in itself, confirm active transmission to humans. However, in urban environments with frequent human–bird interactions, such findings warrant vigilance and preventive awareness.

Continued vigilance is warranted, especially since USUV and WNV often co-circulate in birds and mosquitoes in Europe, sometimes leading to concurrent or alternating outbreaks in humans and horses (16). The lack of avian influenza positives in our study does not eliminate the risk that AIVs pose; indeed, ongoing circulation of various avian influenza strains (including highly pathogenic H5Nx subtypes) in wild and domestic birds across Europe remains a significant public health and veterinary concern (24). The absence of AIV positives in our study could also reflect the limitations of sample size or sampling strategy, although the RT-qPCR assays applied are considered to have high analytical sensitivity and specificity (42). Thus, active surveillance for avian influenza in both wild birds and poultry should continue in our region, even if our three-year dataset did not capture any cases.

The concurrent presence of *C. psittaci* and WNV in our study underscores the diverse zoonotic threats associated with birds in an urban environment. These findings highlight the necessity for ongoing surveillance and an initiative-taking One Health strategy. Monitoring avian populations for pathogens can serve as an early warning system for zoonoses that may eventually affect humans. Given the increasing interaction between humans and birds in urban settings like parks, squares, and even backyard poultry or pet bird keeping, understanding pathogen transmission dynamics is critical for informing effective public health interventions. For *C. psittaci*, public health measures could include educating the public (especially bird owners and individuals feeding wild birds) about psittacosis prevention, implementing controls on feeding wild pigeons in city centres to manage pigeon population density, and ensuring that veterinarians and physicians collaborate to promptly identify and treat cases of psittacosis in humans and birds. For WNV (and other mosquito-borne viruses), an integrated approach involving veterinary authorities, entomologists, and human health agencies is needed.

Our study also underlines the value of the One Health approach, which fosters collaboration across the veterinary, medical, and environmental sectors. In practice, this could mean establishing a surveillance program that simultaneously tracks avian infections (through bird sampling or sentinel species), mosquito populations and viral testing, and human encephalitis cases during the transmission season. Integrated surveillance systems of this kind have proven effective in other countries—for example, Italy’s multi-sector WNV monitoring program (combining bird, mosquito, horse, and human surveillance) has enhanced early outbreak detection and control (41). Strengthening such interdisciplinary surveillance in Bosnia and Herzegovina would likely improve our ability to predict and prevent zoonotic disease outbreaks.

Despite its strengths, this study has several limitations that warrant acknowledgment. First, the sampling strategy was opportunistic and non-random, which may have introduced selection bias regarding species and locations. Additionally, sampling was not specifically stratified by season or vector activity levels, which may have influenced the likelihood of arbovirus detection—particularly in periods of low mosquito abundance. Second, while we detected *Chlamydia* spp. in both pigeons and chickens, species-level identification was only performed for *C. psittaci*. The lack of genetic typing data (e.g., ompA sequencing or MLST) limits our ability to draw conclusions about the diversity and zoonotic potential of the detected strains. Similarly, no molecular characterization of the WNV-positive sample was conducted, and no entomological or human follow-up investigations were undertaken, which limits the interpretation of the broader epidemiological context.

Future studies should address these gaps through a more systematic sampling framework that includes seasonal and vector-based stratification, species-level pathogen typing using molecular methods, and integration with entomological and clinical data, including serological surveys in humans and animals. In particular, the application of multi-locus sequence typing (MLST) for *C. psittaci* and targeted PCR panels for other *Chlamydia* species such as *C. gallinacea* would enhance our understanding of the pathogenic landscape in avian hosts. Our findings support the development of a regional, One Health-based early warning system that integrates avian pathogen surveillance with public health and veterinary networks. Such a system would allow for the early detection of emerging zoonoses and a coordinated response to mitigate risks. Strengthening cross-sectoral collaboration, data sharing, and harmonized surveillance protocols will be essential for achieving this goal in Bosnia and Herzegovina and the wider Western Balkan region.

## 5 Conclusion

The detection of *C. psittaci* and WNV in birds from Sarajevo Canton calls for heightened awareness and preventive actions. Targeted control measures—including improving biosecurity in bird husbandry, reducing risk at the human–bird interface in urban tourist areas, and implementing mosquito control when appropriate—are recommended to mitigate the risks identified. Continued surveillance of wild and captive birds, in coordination with public health monitoring (One Health surveillance), is essential for early identification of emerging zoonotic pathogens. By proactively addressing these issues through a One Health framework, we can better protect both animal and human health in our region. Notably, avian influenza virus (AIV), Chikungunya virus (CHIKV), and Usutu virus (USUV) were not detected in any of the tested bird samples during the study period.

## Data Availability

The original contributions presented in the study are included in the article/Supplementary material, further inquiries can be directed to the corresponding author.
