# Supplementary material for: The monitoring of emergent zoonotic pathogens in wild and captive birds in Sarajevo Canton, Bosnia and Herzegovina
Source: Front Vet Sci. 2025 Jul 23;12:1621094. doi: 10.3389/fvets.2025.1621094 (PMC12327388; doi:10.3389/fvets.2025.1621094)
Supplement: Supplementary file 1 [file Table_1.docx]

Supplementary Material

Supplementary Table 1. Oligonucleotide primer and probe sequences used for molecular detection of *Chlamydia* spp., *C. psittaci*, avian influenza virus (AIV), West Nile virus (WNV), Usutu virus (USUV), and Chikungunya virus (CHIKV).

| **Pathogen** | **Primer/Probe name** | **Sequence (5’-3’)** | **Reference** |
| --- | --- | --- | --- |
| *Chlamydia spp.* | Ch23S-F | CTGAAACCAGTAGCTTATAAGCGG | (Ehricht et al., 2006) |
|  | CH23S-R | ACCTCGCCGTTTAACTTAACTCC |  |
|  | Ch23-P | [FAM]-CTCATCATGCAAAAGGCACGCCG- [TAMRA] |  |
| *Chlamydia psittaci* | CppsOMP1-F | CACTATGTGGGAAGGTGCTTCA | (Pantchev et al., 2009) |
|  | CppsOMP1-R | CTGCGCGGATGCTAATGG |  |
|  | CppsOMP1-P | [FAM]-CGCTACTTGGTGTGAC- [TAMRA] |  |
| Avian Influenza virus | IVA D 161 M-F | AGATGAGYCTTCTAACCGAGGTCG | (Heine et al., 2015) |
|  | IVA D 161 M 1-R | TGCAAAAACATCYTCAAGTCTCTG |  |
|  | IVA D 161 M 2-R | TGCAAACACATCYTCAAGTCTCTG |  |
|  | IVA D 161 M 3-R | TGCAAAGACATCYTCAAGTCTCTG |  |
|  | IVA D 161 M 4-R | TGCAAATACATCYTCAAGTCT CTG |  |
|  | IVA MA-R | [FAM]-TCAGGCCCCCTCAAAGCCGA- [BHQ1] |  |
| West Nile virus | WNproC-F | CCTGTGTGAGCTGACAAACTTAGT | (Linke et al., 2007) |
|  | WNproC-R | GCGTTTTAGCATATTGACAGCC |  |
|  | WNproC-P | [FAM] CCTGGTTTCTTAGACATCGAGATCT-[TAMRA] |  |
| Usutu virus | USU-F | AAAAATGTACGCGGATGACACA | (Cavrini et al., 2011) |
|  | USU-R | TTTGGCCTCGTTGTCAAGATC |  |
|  | USU-P | [FAM]-CGGCTGGGACACCCGGATAACC-[TAMRA] |  |
| Chikungunya virus | CHIKV-F | TGATCCCGACTCAACCATCCT | (Rezza et al., 2007) |
|  | CHIKV-R | GGCAAACGCAGTGGTACTTCCT |  |
|  | CHIKV-P | [FAM]-TCCGACATCATCCTCCTTGCTGGC-[BHQ ] |  |
